# Left-Handed Reformation

**Published:** 1886-09

**Authors:** 


### *LEFT-HANDED REFORMATION.*

Every little while the newspapers give us an account more or less sensational, of some notable conversion or reformation on the part of some member of society, whose prominence adds interest to the event. Sometimes this change of front, not to say heart, is brought about suddenly, and almost miraculously, after the manner of Saul's, with just enough of the romantic to redeem it from the common-place; but more frequently the Evangelist Jones, or Moody, or the Salvation Army is credited with having demolished the stronghold of Satan and put the arch enemy of mankind to flight. Not many months ago Evangelist Moody was credited with captivating a well-known negro minstrel, and the victory was heralded forth as one of great consequence to the whilom delineator of plantation moods and melodies, who, under a stress of conscience, was persuaded to abandon the whole rigmarole of Anglo-African antics for the graver ones of an Evangelical apprentice. And so the gentle voice of Minstrel Brown, which had thrilled many an appreciative audience from behind the foot-lights, was attuned to the well-known attraction of "Hold the Fort," under the leadership of Mr. Moody's musical yoke-fellow.

Mr. Brown was no slouch. A few rehearsals familiarized him with his new role, nor did his modesty stand in the way of his becoming a shining light in the gospel tent; a "card," so to speak, in the enterprise, and a finger-mark for the unconverted. Having been long accustomed to face an audience, and to deal with rather tough conundrums, Mr. Brown naturally enough looked forward to the time when he too should be invested with the cloth. He required very little coaching. Indeed he was surprised at himself to find how readily he apprehended doctrinal points. Not that there was any near resemblance between negro minstrelsy and theology, but his long experience in burned cork had somehow accustomed him to deal off-hand, with the knottiest kinds of problems. His reasoning was something after this fashion: What couldn't be done in six days probably wasn't worth doing at all, and why not knock off for a day after winding everything up and seeing it move on. At first blush he had a little misunderstanding with the enigma of three in one, but this grew out of a professional alertness, for he disliked of all things being *sold*, and never allowed himself to be caught on the wrong side of a laugh if he knew it. He made open confession that to his mind nothing could be more unique than the atonement. It saved a world of trouble in looking after individual cases, and if a fellow hasn't time to atone for himself owing to previous engagements, why shouldn't some one else do it for him if he wants to, all in a bunch with the rest. It's all a matter of taste and *free will.* Only once was Mr. Brown fairly driven into a corner. It was on the woman question as connected with original sin. Forgetful of himself in a dream of his former accomplishments as an end man, in true negro dialect, he "guv it up."

After this going over, honors were easy, and in due course of time the distinction of Reverend was conferred by the laying on of hands, instead of burned cork.

The next thing on the catalogue of expectation was a *call.* It came about in good time, for a real simon-pure reformed negro minstrel was sure to be a paying investment in any well-organized Christian society.

Strangely enough it never occurred to any of the brethren that a good negro minstrel is always preferable to an indifferent preacher, although dulness seems to be less objectionable in the pulpit than almost anywhere else. This is, perhaps, because we have become accustomed to it. The point we wish to make is that there are preachers who would undergo genuine reformation if converted into good negro minstrels, and their lives devoted to furnishing the public with rational amusements.

There was a good deal of curiosity to see if not to hear the new minister, who had so magnanimously laid aside the banjo for the harp. The church was Presbyterian—old school—with a fair sprinkling of Scotch among the parishioners. But this suited the Rev. Mr. Brown's theological bent, and right lustily he rang the changes on the severe old creed, presenting anything but a sprightly outlook to the unredeemed and unregenerated. At times he fairly ran riot with his theme, setting up one dummy after another, as a centre of attack, and tumbling them over with the skill of an oratorical athlete. Conundrums! Well yes, he handled a few somewhat after the old style, and looked about a little surprised when nobody audibly gave them up. The leathery old elders nudged one another between cat-naps, as much as to say, "we've made no mistake this time." It was evident the new minister had entered upon his first campaign determined to succeed or know the reason why. In the figurative terms of the Western cowboy, he had resolved to make a "spoon or spoil a horn," and now he was giving his spoon a first trial by stirring up things promiscuously. And why shouldn't he succeed? If anybody could swoop down upon some beggarly human frailty and cover it all over with invective, the Rev. Mr. Brown could. Then, almost in the same breath, as if touched by the sweep of an angel wing, his voice would modulate itself to the sweet persuasiveness of a father, calling home to the parental fold some misguided lamb, and then again would he teasingly titillate some old doctrinal scab,

> "As if divinity had catched
> The itch on purpose to be scratched."

If his sermons lacked method, they were as choke full of matter as a carbuncle or a boil.

For a time everything was lovely. Then little by little the faintest shade of discontent began to exhibit itself, and take root, and grow, till like a dark cloud, it settled down upon the church, and neither prayers nor incantations availed for its dissipation. It was not so much the pastor's rendering of "the law and the prophets" that furnished the grounds of complaint, as his way of putting things. It was gravely hinted that the old formality of *thee* and *thou* might sometimes be used with propriety, especially in addressing the throne of grace; but the reformed negro minstrel thought otherwise, and squared himself away upon his dignity, which, among gentlemen of the cloth, is significant of a dogged determination to fight it out. Besides he was not to be bullied into compliance, and so in his prayers and supplications the new pastor still persisted in using the more familiar forms of expression, such as "you God and your Son."

The church is vacant now. The Reverend-Minstrel has left, but he carried with him two sets of resolutions—one of disseverance or dismissal and another of commendation. Perhaps, after the manner of a leading character in "Festus," the deacons were induced to compromise by thanking God that he was no worse, and resolved accordingly.

*Moral*—"Shoemaker, stick to your last."

